# Assessing the Acceptability of a Peer Mentor Mother Intervention to Improve Retention in Care of Postpartum Women Living with HIV

**DOI:** 10.1089/heq.2019.0027

**Published:** 2019-07-01

**Authors:** Yetunde Akinde, Allison K. Groves, Hervette Nkwihoreze, Erika Aaron, Gregg Alleyne, Charmaine Wright, John Jemmott, Florence M. Momplaisir

**Affiliations:** ^1^ Department of Community and Prevention, Dornsife School of Public Health, Drexel University, Philadelphia, Pennsylvania.; ^2^ Division of Infectious Diseases and HIV Medicine, School of Medicine, Drexel University, Philadelphia, Pennsylvania.; ^3^ Philadelphia Department of Public Health, AIDS Activities Coordinating Office, Philadelphia, Pennsylvania.; ^4^ Department of Obstetrics and Gynecology, School of Medicine, Drexel University, Philadelphia, Pennsylvania.; ^5^ Department of Medicine, Center for Special Health Care Needs, Christiana Care, Wilmington, Delaware.; ^6^ Department of Psychiatry, Perelman School of Medicine, University of Pennsylvania, Philadelphia, Pennsylvania.

**Keywords:** adherence, HIV/AIDS, HIV care continuum, maternal health, retention in care

## Abstract

**Background:** Many women living with HIV (WLWH) experience poor postpartum retention in HIV care. There are limited evidence-based interventions in the United States aimed at increasing retention of WLWH postpartum; however, evidence from low-resource settings suggest that women who receive peer mentoring experience higher retention and viral suppression postpartum.

**Methods:** We conducted 15 semistructured interviews with pregnant or postpartum women from an urban U.S. clinic to assess factors influencing maternal adherence to antiretroviral therapy (ART) and retention in HIV care. We then assessed the acceptability of a peer intervention in mitigating barriers to sustain adherence and retention in care postpartum. Interviews were audio taped, transcribed, and analyzed. Codes were developed and applied to all transcripts, and matrices were used to facilitate comparisons across different types of participants.

**Results:** Participants included low-income black and Hispanic women with a mean age of 31 years (range 22–42). Social support and concern for infants' well-being were strong facilitators for engaging in care. Psychosocial challenges, such as stigma and isolation, fear of disclosure, and depression, negatively influenced adherence to ART and engagement in care. Regardless of their level of adherence to ART, women felt that peer mentoring would be an acceptable intervention to reinforce skill-related ART adherence and sustain engagement in care after delivery.

**Conclusion:** A peer mentor mother program is a promising intervention that can improve the care continuum of pregnant and postpartum women in the United States. Messaging that maximizes maternal support and women's motivation to keep their infant healthy may leverage retention in care postpartum.

## Introduction

Women living with HIV (WLWH) experience poor viral suppression during labor and delivery and particularly in the postpartum period.^1,2^ An elevated viral load increases the risk of perinatal transmission of HIV and can lead to maternal progression to AIDS. Although the United States is reaching elimination goals for perinatal transmission of HIV,^3^ racial disparities persist with perinatal transmission being 10 times higher among blacks compared to Hispanics and whites.^4^ Poor retention in HIV care postpartum can lead to poor maternal health and can compromise pediatric health. Despite this period of vulnerability, there is a lack of evidence-based interventions aimed at improving retention and viral suppression postpartum for U.S. women.^5^

Peer mentors are WLWH who provide education and support and enhance coping skills of pregnant women with a new or established diagnosis of HIV.^6,7^ Published studies from sub-Saharan Africa show that WLWH who receive peer mentor education and support have higher adherence to antiretroviral therapy (ART), improved maternal health and well-being and infant HIV testing, and a lower risk for perinatal HIV transmission.^7–9^ Peer interventions range from those which only target HIV to others which address challenges mothers face in the postpartum period (including but not restricted to HIV).^10^ The implementation and use of such an intervention in the United States has the potential to improve maternal HIV outcomes. The purpose of this qualitative study is twofold as follows: (1) to better understand psychosocial and structural factors influencing engagement in care and adherence to ART during pregnancy and the postpartum period and (2) to assess the acceptability of a peer mentor intervention in serving the needs of pregnant and postpartum women in a resource-rich setting. Based on the needs expressed by women, as well as their perspectives on the acceptability of the proposed peer-based intervention, we aim to refine educational materials for a peer-based intervention to foster sustained adherence to ART and retention in HIV care during pregnancy and postpartum.

## Methods

### Setting and study participants

This study was conducted at a comprehensive HIV and obstetrics and gynecology care clinic in Philadelphia, Pennsylvania. Women were recruited during routine prenatal or postnatal care appointments for in-depth individual interviews. Inclusion criteria for the study were as follows: (1) self-identify as black and/or Hispanic, (2) age 18 years or older, (3) with a known diagnosis of HIV, (4) and were in their first or second trimesters of pregnancy, or ≤6 months postpartum. Institutional Review Board approval from Drexel College of Medicine was obtained before the start of the study. We purposively sampled women from both pregnancy and the postnatal period to better understand needs specific to each time period.

### Interview procedures

We conducted in-depth semistructured interviews with fifteen women until we reached code saturation (i.e., no new barriers or facilitators)^11^; specifically, we conducted eight interviews with pregnant women and seven interviews with postpartum women meeting inclusion criteria. The interviews were typically 45–60 min in length and were done individually in a private room. The first part of the interview examined psychosocial and structural factors influencing engagement in care and adherence to ART during pregnancy and the postpartum period. The second part of the interview focused on the acceptability of a peer mentor program and examined comfort level, interest level, and whether the program would be responsive to the needs of the participants.

### Data analysis

Interviews were audio recorded and transcribed verbatim with personal identifiers removed.^12,13^ A codebook was created using deductive and inductive codes developed after thoroughly reviewing interview transcripts.^14^ As the analysis progressed, new themes emerged, and the codebook was refined and extended. We extensively reviewed data and analyzed them through the following steps: (1) Coding: we categorized our data by developing and applying inductive and deductive codes to all transcripts using Dedoose software.^15,16^ Deductive codes were based on field guide instruments and key research questions. Inductive codes were derived through team meetings to capture emergent themes. (2) Matrices: we used matrices to further facilitate comparisons across different types of participants (e.g., those with mental health issues vs. those without mental health issues).^12^ (3) Memos: we created detailed memos that highlight key components of the intervention that are needed to be successful.

## Results

Demographics of participants are summarized in Table 1. Women were predominantly black (93%), low-income women, with a mean age of 35 years (range 23–42). All women were on ART at the time of the interview.

**Table 1.** Participant Demographics

*N*	15
Age, mean	31 (22–42)
Years with HIV, mean	10 (1–24)
Race, *n* (%)	
Black	14 (93)
Black and Hispanic	1 (7)
Education, *n* (%)	
Less than HS	3 (20)
HS graduate	5 (33)
Some college	7 (47)
Income (thousands), *n* (%)	
<10	11 (73)
>10	3 (20)
No response	1 (6)
Marital status, *n* (%)	
Single	8 (53)
Cohabitating/married	7 (47)
Mental health, *n* (%)	
Depression and/or anxiety	11 (73)
Other	3 (20)
None	1 (7)
Disclosure to partner, *n* (%)	
Yes	12 (80)
No	3 (20)
Number of living children, *n* (%)	
0	1 (7)
1–3	11 (73)
3–5	3 (20)
Employment status, *n* (%)	
Unemployed	8 (53)
Part-time	4 (27)
Full time	3 (20)

HS, high school.

Our analysis revealed major barriers and facilitators faced by WLWH. Starting with facilitators, concern for infant welfare and social support positively influenced women's ability to engage in care. Conversely, psychosocial challenges such as stigma and isolation, fear of disclosure, and concurrent depression and/or anxiety negatively influenced care engagement. Furthermore, women experienced structural challenges related to housing stability, employment, and health insurance. The combination of these psychosocial and structural challenges negatively impacted women's care engagement and retention in HIV care. Women felt that a peer mentor program would be an acceptable intervention for navigating key challenges during the postpartum period.

### Factors influencing adherence to ART and engagement in HIV care during pregnancy and postpartum

#### Prevention of perinatal HIV

For all women, the biggest motivator to engaging in care and adhering to ART was prevention of perinatal transmission of HIV. The fear of this risk drove women to attend HIV care visits and adhere to their ART. Women who were not adherent to their ART before pregnancy changed their behavior during pregnancy: “I just started taking my medicine while I was pregnant. I never took medicine in my life.” During pregnancy, women put their children's needs above their own and overcame challenges associated with ART adherence to protect their infant: “I got that motivation that everything I do is for this baby, not for me, it's for the baby because kids come first so I have to put my baby first before I put myself. So that's how I keep my motivation going.” Those who were adherent and in care before pregnancy reinforced their care seeking behavior: “Meaning as though I have a baby now, it would make me want to go [to my appointments] even more just to make sure my baby is healthy, and I'm still healthy, and we are on the right path.”

#### Social support

Women who received support regarding their HIV status from family members, partners, and friends reported enhanced coping skills, which helped to have consistent care seeking behavior during pregnancy and postpartum. Women particularly found their mothers to be key providers of emotional and instrumental support regarding their HIV status and childcare. “She's [my mother] been supportive since I told her [about my HIV status] since day one; so like my mom, she's my backbone. Everything I go through, I talk to my mom. So it's like my mom, she's been more of my support than anything through this whole entire thing.” In addition to mothers, some partners were also key providers of support. “It's actually easier considering that I am not doing things by myself. Now if I was doing things by myself, I probably would be overwhelmed and stressed out. Like he [my partner] didn't miss a doctor visit the whole time I was pregnant. Even now, he doesn't miss anything so I am kind of glad I'm not going through the circumstances by myself and that he was able to give me his insight on it and explain to me that it wasn't a death sentence.” Some partners also helped women with reminders to take their ART and sometimes attended their medical visits with them. “If he is not working, he is there. He will remind me for the most part, like ‘did you take your medicine today? Did you take your medicine?’ [if] I need him to babysit or watch the kids, he will do that for me. If I need carfare to get here, he will make sure I have carfare to get here and get back, so he is a really big help.”

#### Stigma and isolation

From the onset of diagnosis, many women experienced internalized stigma and endorsed negative beliefs and feelings associated with HIV. For example, one participant said, “…the stigma alone with this HIV/AIDS, all that comes with it is such a disgusting disease, dirty people get that [crying], you know, all of that, I wanted to have cancer, I wanted to have cancer instead of this.” Many women reported ongoing challenges with stigma, which led to self-isolation with women distancing themselves from family and friends. Many women had no personal connections to other WLWH, which made it harder to receive support from individuals with shared experiences. When discussing her reaction after being diagnosed with HIV, one woman who was diagnosed during pregnancy said, “once I found that out, I didn't want to be bothered with nobody so actually I didn't even hang out with nobody after that. I just stayed in the house looking out the window…I just kept trying to think that why did this happen to me? Am I going to die? Is my baby going to die? It was just like a lot going on.” Furthermore, women were aware of the negative perceptions surrounding individuals living with HIV and tried to find ways of protecting themselves from being stigmatized. For example, many women were uncomfortable with having their ART prescription bottles in their houses and often resorted to hiding their pills. “I don't want everybody in my business so like my medicine I don't have it in the actual pill bottle I will put it in like an Ibuprofen bottle…. when I get my medicine, I tear my name off of it and I just put it in a different container.” Having to hide their ART pill bottles, particularly postpartum when family and friends came to visit and when they were consumed with infant care, was disruptive to ART adherence. Some women would even forget where they hid their ART pill bottles.

#### Disclosure

For a lot of women, feelings of isolation were further compounded by challenges with disclosure. The majority of women expressed struggling with disclosing their HIV status; they worried about how disclosure would affect the dynamics of their relationship with their partner. For example, one participant said “…I don't want to have to tell him that I have it [HIV] and he looks at me totally different. It's going to be a problem for me because you should love me for me.” During the pregnancy period, receiving emotional, instrumental, and financial support was increasingly important for women. Some women contemplated sharing their HIV status with their partner after the news of pregnancy but again the fear of losing their partners and the fear of rejection kept them from doing so. One participant said, “it's like how do you tell a man that you're pregnant by that you have HIV? That's the thoughts that's going on in my mind. It's like I want to tell him, then again, it's like I don't want to tell him because I didn't know how he's going to accept it or how he's going to reject to it.” Navigating the disclosure process was even more challenging during pregnancy as disclosure not only affected both partners but also the unborn child. For some women who chose to disclose, the relationship dynamic was negatively impacted. One participant said, “after I disclosed, we kept on arguing and stuff like that. You know, about the baby, about HIV. He's being paranoid because I'm HIV positive. He thinks his baby is going to have it and I keep on telling him that as long as I take my medicine and I take care of myself, the baby's not going to get it.”

### Depression and/or anxiety

A large proportion of our participants had a past or current diagnosis of depression and/or anxiety and experienced exacerbation of their symptoms during pregnancy and postpartum. Women often experienced worsening feelings of sadness, guilt, and anxiety compounded with the challenges of living with HIV. For some women, depression was a significant factor in their ability to consistently engage in care. When discussing factors that affect regular attendance to medical visits, one participant said: “What would stop me [from going to clinic]? Maybe my depression. Nothing else. Nothing else would get in the way of anything. I'd just be my depression. Days I don't want to get out bed, that's it. Other than that, I'm going to go. I'm going to go, I'm going to go. It's just my depression takes over me.” During pregnancy, many women stopped taking their antidepressants because of the fear of teratogenicity often without consulting with their medical provider.

### Adjustment associated with infant care and responsibilities

Women's motivation and ability to be adherent to their ART and attend their medical appointments weaned during the postpartum period in the face of competing priorities related to childcare. For many, adjusting to having a newborn was the biggest challenge: “I gotta do it all over again. Getting up, late nights, and making bottles, and feeding, changing diapers, and um, in the daytime feeding them or taking them to their first appointments, and um, trying to get enough sleep.” The adjustment process was even tougher for women with other young children. Many expressed feelings of anxiety about providing the best care for their newborn and other children. “I know like for me, it's not only about me and about my kids but it's still stressful taking care of my son and now I have another child that's coming. I have my doubts sometimes.”

### Structural challenges faced by WLWH

In addition to psychosocial challenges, women also faced ongoing structural challenges related to housing instability and lack of employment. Within our sample, the most common structural challenge was housing instability. Some of the women were homeless while pregnant and had to be in shelters, sometimes with their young children. Other women resolved to moving in with friends and relatives or living in poor and cramped housing conditions. When discussing her living situation, one participant said, “I sleep in the living room on my couch you know and we need our beds, especially [my son] because he keeps on having nightmares and stuff.” In addition, women also reported facing difficulties with securing consistent employment. More than half of the women were unemployed at the time of the study, with a majority receiving welfare assistance. Among the women who were employed, some planned on cutting back from full-time to part-time employment, while others mentioned planning to stop working all together due to lack of adequate maternity leave benefits with their current employers.

### Syndemic effect of psychosocial and structural factors

Often, women experienced psychosocial and structural stressors simultaneously, and they acted synergistically to negatively affect women's ability to engage in care. One pregnant woman with a history of depression, in a violent relationship, had just left her partner and went to a shelter with her 4-year-old daughter, described how she was struggling to get food for herself and her daughter. She explained how stressors associated with lack of income, housing and food instability, and being pregnant affected her mental health and well-being: “It was like I am pregnant, shelter, and I am still working. And I still don't have no money…No. Now, I am going to have the baby which is more stress, more mental problems. So, it was just a whole lot on my last daughter. It was like, I didn't want to do nothing. I didn't want to go outside [to get food]. I didn't want to go nowhere.”

### Acceptability of peer mentor program

All the women interviewed believed a peer mentor program would be an acceptable intervention for improving adherence to ART and retention in HIV care during the postpartum period. Despite the differing needs of women, the core need for all was in wanting support during the postpartum period. “I think it's a good idea to have [a peer] because most moms that are out there and have HIV and are pregnant are scared. They even feel bottled, they feel shelled, they feel like don't nobody understand them, everybody's going to look at them differently.” Women found the idea of a peer mentor intervention to be acceptable due to the shared experiences between them and the peer mentors. Because mentors are also mothers living with HIV, women found them to be more credible in providing support and counseling: “Honestly, for me, like I don't know other HIV-positive women and HIV-positive pregnant mothers but it [the peer intervention] almost makes you feel like you are not so alone. There's somebody that's in a similar situation you know. I mean you feel isolated enough…but just to have someone in similar circumstances and dealing with similar things.”

While some women reported having support systems consisting of partners, family members, and friends, many felt that there was a gap in the support they were currently receiving, mostly because supportive individuals were not able to fully understand their experiences of living with HIV the same way a peer would: “…it's harder to talk about how I'm feeling so I figured if there's a community of women that has it [HIV], I'd be easier and open to express my feelings. Sometimes I'm like who can I talk to? I can't talk to my mom, I can't talk to my sister because they don't know, they don't experience it.” They felt comfortable meeting with the peer in clinic and some in their home. They reported that face-to-face meetings, phone calls, and text messages were appropriate ways of communication.

In addition, women stated that peers could help with coping skills, particularly for women with a new diagnosis of HIV during pregnancy. They also thought that peers could help with HIV education and education on other aspects of living with HIV. For example, they felt that it would be beneficial to have the peer explain the advantages and disadvantages of disclosure, identify individuals around them to whom they can disclose, and prepare them for disclosure conversation.

## Discussion

Women faced many challenges that interfered with their ability to be adherent to their ART and be retained in HIV care in the postpartum period; some of these challenges were personal, such as stigma and depression, and some were structural, such as housing instability and lack of employment. These challenges occurred in the context of demands associated with caring for a newborn, sometimes with limited social support. Study participants thought that the peer mentor intervention would be an acceptable measure to mitigate challenges in the postpartum period. Because of the shared experience of being HIV positive and having experienced pregnancy while HIV positive, women felt that peers could be a credible source to provide HIV-related education and support during the perinatal period.

Many of the challenges that women reported in the qualitative interviews regarding ART adherence and retention in HIV care postpartum have been previously described in low- and high-resource settings. Despite geographic and cultural differences across these settings, challenges seem to be very similar. According to a systematic review of the literature, drivers for ART adherence for WLWH in sub-Saharan Africa include a strong commitment to pediatric health and spousal involvement in HIV treatment.^17^ Barriers include stigma, nondisclosure to their partner, challenges with practical demands of ART adherence, and a poor understanding of perinatal transmission of HIV.^17^ Another systematic review from low-resource countries focusing on structural-level factors associated with maternal adherence to ART and care engagement described barriers related to access to services, care coordination across health systems, and gaps in provider training,^18^ which, to some extent, also apply to the U.S. context. Interviews of pregnant or postpartum women in the Southern United States showed that women's strongest motivator to stay engaged in care during pregnancy was to keep their children healthy; barriers included issues related to transportation and scheduling conflicts with work and childcare transportation and experiences of institutionalized stigma.^19,20^ Given shared concerns of WLWH despite geosocial differences, adapting a peer mentor program that has been successful in improving HIV maternal and pediatric outcomes in low-resource settings, to meet the needs of pregnant or postpartum women in the United States, seems promising.

The use of peer mentors in Sub-Saharan Africa has been shown to improve postpartum retention in HIV care in retrospective studies and in randomized control trials. A cluster randomized controlled trial in KwaZulu-Natal, South Africa showed strong benefits of peer mentorship for infant outcomes (exclusive breastfeeding and increased weight gain) and moderate benefit for postpartum retention.^21^ In this study, eight clinics were randomized for pregnant WLWH to receive either a Standard Care condition (4 clinics; *n*=656 WLWH) or an Enhanced Intervention (4 clinics; *n*=544 WLWH) with a peer mentor. The investigators found that 63% of women in the intervention arm had at least one postpartum visit at 6 months compared to 45% in the control arm (*p*=0.13). Women in the intervention arm were also less likely to express depressive symptoms (*p*<0.01). In Malawi, postpartum retention was significantly better for facility-based (80%) and community-based (83%) peer mentors compared to standard of care (66%).^9^ Facility-based mentors met women in the clinic, whereas community-based mentors met women in their home. Other nonrandomized studies showed significant improvement in postpartum retention in HIV care.^22,23^ Core to the success of peers is training (and retraining when appropriate), outcome specific scope of work, supervision, and the use of specific educational materials for HIV education.^6^

Limitations for our study include sampling bias since interviews were conducted among participants who showed up for clinic visits and were engaged in care. Therefore, we were not able to capture the perspectives of women who disengaged from care. Even though acceptability was high, we lack information on the feasibility of a peer intervention in perinatal HIV clinics; advance planning on how to best integrate the peer role within the clinical care team needs to occur. Finally, educational materials for the peer to use need to be developed based on the themes that emerged in our study and the existing literature.

In conclusion, we found a peer mentor intervention to be highly acceptable among pregnant and postpartum WLWH. In addition to providing education, peer mentors can provide emotional and social support and help women address challenges specific to the perinatal period. Peers can capitalize on their own experience and women's motivation to protect their children from HIV during pregnancy to sustain ART adherence and care engagement postpartum. The feasibility and effectiveness of a peer mentor intervention to improve maternal retention in care and adherence to ART remain to be tested.

## Abbreviations Used

ART: antiretroviral therapy
WLWH: women living with HIV
